# Rumen Development of Artificially-Reared Lambs Exposed to Three Different Rearing Regimens

**DOI:** 10.3390/ani11123606

**Published:** 2021-12-20

**Authors:** Hitihamy M. G. P. Herath, Sarah J. Pain, Paul R. Kenyon, Hugh T. Blair, Patrick C. H. Morel

**Affiliations:** 1 School of Agriculture and Environment, Massey University, Private Bag 11222, Palmerston North 4442, New Zealand; S.J.Pain@massey.ac.nz (S.J.P.); P.R.Kenyon@massey.ac.nz (P.R.K.); H.Blair@massey.ac.nz (H.T.B.); P.C.Morel@massey.ac.nz (P.C.H.M.); 2 Department of Livestock Production, Faculty of Agricultural Sciences, Sabaragamuwa University of Sri Lanka, Belihuloya 70140, Sri Lanka

**Keywords:** milk replacer, neutral detergent fibre, papillae development, pellet composition, rumen site, ultrasound scan, volatile fatty acids, age at weaning

## Abstract

**Simple Summary:**

Young ruminants possess an undeveloped rumen when first born. Encouraging good rumen development in early life is vital for young ruminants as they transition to a solid feed diet, to ensure optimum growth post-weaning. This study aimed to investigate the effect of three different rearing regimens on rumen development in lambs reared artificially. Weaning of lambs at 42 d of age improved rumen fluid n-butyric content and also resulted in a thicker rumen dorsal wall. Feeding lambs pellets with low fibre and weaning them early increased rumen fluid n-valeric content. Papillae width, density, and rumen wall muscle thickness were affected by rearing treatment. Empty rumen weight, rumen volume, papillae development, and rumen fluid iso-butyric and iso-valeric content had positive associations with dry matter intake and nutrient intakes from solid feed. The majority of volatile fatty acids in the rumen fluid had a positive association with papillae height on the rumen dorsal wall. These results suggest that lamb diet and age at weaning influenced rumen function and physical development. Further studies examining how rumen microbial composition and rumen gene expression are influenced by rearing regimens are required.

**Abstract:**

The objective of this study was to examine the effect of three different rearing regimens on rumen development in lambs reared artificially. Romney ram lambs were randomly allocated to one of three treatments: commercial milk replacer fed to 57 d of age and high fibre concentrate pellets (HFP57); commercial milk replacer, high fibre concentrate pellets, and early weaning from milk replacer at 42 d of age (HFP42); high protein milk replacer from 2–16 d of age followed by commercial milk replacer, low fibre concentrate pellets, and early weaning from milk replacer at 42 d of age (LFP42). Lambs were slaughtered at 57 d of age. Volatile fatty acid content in rumen fluid at slaughter was analysed and rumen tissue samples were collected for histological examination. The rumen n-butyric content was greater (*p* < 0.05) in both LFP42 and HFP42 treatment lambs compared to HFP57 lambs. The n-valeric content was greater (*p* < 0.05) in LFP42 lambs compared to both HFP57 and HFP42 treatment lambs. Thickness of the rumen dorsal wall determined by ultrasound scanning at 49 d was greater (*p* < 0.05) in both HFP42 and LFP42 lambs compared to HFP57 lambs. There was an interaction (*p* < 0.05) between treatment and site of rumen tissue sampling on papillae width, density, and rumen muscular layer thickness. Collectively, early weaning and the provision of a low fibre pellet leads to improved rumen function and physical development.

## 1. Introduction

At birth, the rumen is anatomically and physiologically underdeveloped in lambs [1]. The development of the pre-stomach in a young ruminant undergoes three phases: the non-ruminant phase (birth to 3 weeks), the transition phase (from non-ruminant to ruminant stage), and the ruminant phase [2]. Appropriate rumen development is an important physiological change that facilitates alterations in nutrient metabolism and absorption [3], and consequently, it influences the early growth of young ruminants. A young ruminant’s dietary regimen is the key factor affecting solid feed intake, establishment of rumen microflora, rumen fermentation, and rumen papillae development, all of which contribute to nutrient digestion and absorption [4].

Unrestricted feeding, or a higher quantity of milk replacer feeding, reduces starter solid feed intake of calves [5,6]. This can delay rumen development and reduce performance during the transition period to a pasture-based diet. In contrast, early milk weaning or restricted milk feeding increases solid feed intake in lambs [7]. Early solid feed intake results in earlier establishment of rumen fermentation capacity [8] and greater utilisation of nutrients from solid feed [6]. This helps facilitate a smoother transition from the non-ruminant to ruminant stage, with less impact on post-weaning growth in young ruminants [9].

In general, fibre level in a diet affects the mass and volume of the rumen rather than papillary development [10]. Further, it has been reported that digestion of a roughage-based diet in the rumen does not provide adequate butyrate, which is required for rumen papillae development [11]. Grain-based concentrate diets result in higher butyrate through rumen fermentation, which helps stimulate papillae development in young ruminants [12]. Thus, a combination of a nutrient-balanced concentrate diet with fibre should improve overall rumen development. However, increased fibre level in a concentrate diet can reduce feed digestibility, especially in the early life period of young ruminants who have an underdeveloped rumen [13]. Thus, early weaned lamb rearing systems highlight the importance of feeding highly digestible concentrate feed with adequate fibre before milk weaning occurs, to optimise lamb growth and maximise cost effectiveness [14,15].

There are knowledge gaps regarding the effect of both grain-based pellet fibre level and early weaning on lamb rumen development. This study aimed to investigate the effect of three rearing regimens on rumen development of artificially reared lambs. The three rearing regimens are different combinations of pellet fibre level, milk replacer composition, and age at weaning [15].

## 2. Materials and Methods

### 2.1. Animal Management

The experimental rearing regimens and lambs utilised for this study have been previously described by Herath et al. [15]. The experiment was conducted with lambs between 2 and 57 d of age, as described below.

Twenty-seven twin-born Romney ram lambs were allowed to suckle from their dam for the first 24 h after birth before one lamb per set was randomly selected for the study [15]. The lambs were then individually penned and randomly allocated to rearing treatments. The three rearing treatments were (i) commercial milk replacer fed to 57 d of age plus high fibre concentrate pellets (HFP57, *n* = 9); (ii): commercial milk replacer, high fibre concentrate pellets, and weaning from milk replacer at 42 d of age (HFP42, *n* = 9); (iii) high protein milk replacer from 2–16 d of age, followed by commercial milk replacer, low fibre concentrate pellets, and weaning from milk replacer at 42 d of age (LFP42, *n* = 9). All lambs were provided milk replacer at 2.1 times their maintenance energy requirement [15], considering maintenance energy requirement (ME_m_) as 0.40 MJ/kg live weight (LW)^0.75^ d^−1^ [16]. The crude protein (CP) and metabolisable energy (ME) contents (expressed as fed basis) of commercial milk replacer powder were 263 g/kg and 21.8 MJ/kg, respectively (Milligans Feed Ltd., Oamaru, New Zealand). The high protein milk replacer powder was a mixture of the commercial milk replacer powder and powdered milk protein concentrate (Fonterra, Auckland, New Zealand), mixed at a ratio of 80:20. The CP and ME contents of high protein milk replacer powder were 324 g/kg and 20.8 MJ/kg, respectively.

Milk replacer and high protein milk replacer were mixed with warm tap water at a ratio of 1/4 (w/w). Bottle feeding of lambs was done five times daily up to two weeks of age, then four times daily up to milk weaning phase at day 38–42 for HFP42 and LFP42 lambs, and until the end of the experiment for HFP57 lambs (57 d).

Lambs were offered either high fibre concentrate pellet (69 g/kg acid detergent fibre (ADF) and 209 g/kg neutral detergent fibre (NDF), for HFP57 and HFP42 lambs) or low fibre concentrate pellet (44.40 g/kg ADF and 117 g/kg NDF for LFP42 lambs) ad libitum from 4 to 57 d of age (Table 1). The lambs had free access to water at all the times. Lucerne chaff contained 115 g/kg CP and 7.14 MJ/kg ME (Oaklane Stables Premium Chaff, Hawkes Bay, New Zealand).

Lambs in HFP42 and LFP42 treatments were provided 50% of their milk allowance during the 5 d of milk weaning phase (38–42 d of age), while HFP57 lambs were given their full milk allowance until 57 d of age. The HFP42 and LFP42 lambs were bottle-fed twice daily during the milk weaning phase and fully weaned from milk at 42 d. During the milk weaning phase, lambs in HFP57 and HFP42 were offered ad libitum access to high fibre concentrate pellets, while LFP42 lambs were offered ad libitum access to low fibre concentrate pellets. All the lambs were provided 40 g/d of lucerne chaff through the day 38–57 period. Pellet, milk replacer, and lucerne chaff intakes were recorded daily. All lambs remained in the study until 57 d of age.

### 2.2. Ultrasound Scanning of Rumen

**Figure 1:**
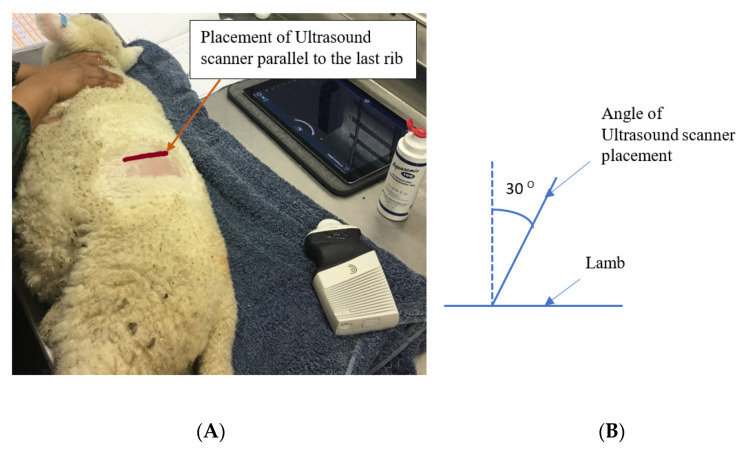
The site (**A**) and angle (**B**) of positioning of Ultrasound scanner to capture rumen images.

Ultrasound scanning of each lamb’s rumen was performed at experimental weeks five (36 ± 1.2 d of age) and seven (49 ± 1.2 d of age). Scanning was undertaken with the lamb lying on its right side, using a Clarius portable ultrasound scanner with a 3.0–7.0 MHz convex transducer and maximum penetration depth of 20 cm (Clarius, Gilmore way, Burnaby, Canada). The wool was clipped from the area on the dorsal left side, just caudal to the last rib, to the end of the transverse process of the last lumbar vertebrae. The rumen was examined caudal to the last rib by placing the ultrasound scanner parallel to the last rib with approximately a 30° angle from the zenith (Figure 1), after application of transmission gel (Aquasonic, Parker laboratories Inc., Fairfield, New Jersey, NY, USA). Images were then captured and the thickness of the rumen wall was measured (9 measurements per lamb) by FIJI imageJ software (National Institutes of Health, Bethesda, MD, USA) [17].

### 2.3. Slaughter

Lambs were slaughtered at 57 d of age regardless of their live weight. The lambs were slaughtered via captive bolt after being fasted for approximately 12 h, exsanguinated, skinned, and eviscerated. Rumen fluid samples were collected into cryovials with phosphate buffer saline (PBS) immediately after opening the rumen, for the analysis of volatile fatty acid (VFA) content. All rumen fluid samples were stored in liquid nitrogen immediately after collection and then at −80 °C until further analysis.

Tissue samples, approximately 1.5 cm × 1.5 cm, were collected at slaughter from each lamb, from the rumen dorsal and ventral walls and placed in histology cassettes with a tissue sponge. The tissues were fixed in 4% paraformaldehyde for 24 h and transferred to 70% ethanol solution until blocks were prepared for histology slides.

### 2.4. Analysis of Rumen Fluid Samples

Rumen fluid samples were analysed at the Nutrition Laboratory, Massey University, Palmerston North, New Zealand. Volatile fatty acid (VFA) content of rumen fluid collected at slaughter was analysed by gas chromatography as described by Sukhija and Palmquist [18]. Briefly, rumen fluid samples were suspended in toluene and fatty acids were methylated by using methanolic hydrochloride (a mixture of methanol and acetyl chloride) in culture tubes. Samples were vortexed and heated at 70 °C for methylation for 2 h. After methylation, samples were cooled on ice and potassium carbonate and toluene were added. Samples were vortexed and centrifuged at 2500 rpm for 7 min at room temperature to separate the solvent layer containing methyl esters and the aqueous layer. The VFA content was determined by Shimadzu GC-2010 Plus Gas Chromatograph (Shimadzu, Kyoto, Japan) equipped with a Supelco^TM^-2560 Capillary Column (100 m × 0.25 mm × 0.2 μm film thickness). The acetic, propionic, n-butyric, iso-butyric, iso-valeric, n-valeric, and n-caproic acid contents were determined.

### 2.5. Histology

**Figure 2:**
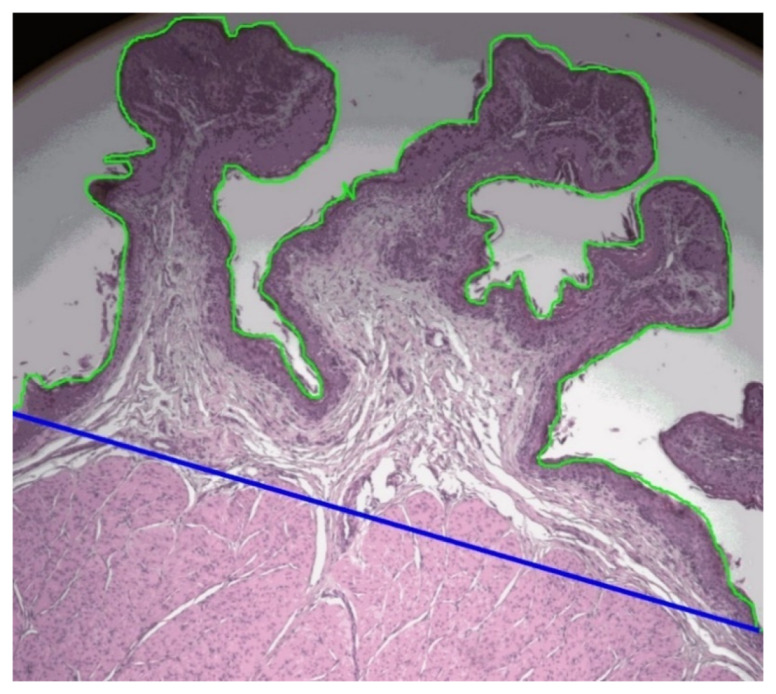
The length of papillae outer boundary of the stratum corneum (green line) and the respective straight length of rumen tissue (blue line) measured in dorsal and ventral sites of rumen by FIJI ImageJ software, to calculate the ratio between length of papillae surface/straight length of rumen tissue (PSL/STL) of lambs.

Histology slides of rumen dorsal and ventral tissue samples were prepared by the Histopathology Laboratory of the School of Veterinary Science, Massey University, Palmerston North, New Zealand. Rumen tissue samples were dehydrated overnight in graded alcohol and embedded in wax (Histostar Embedding, Thermo Fisher Scientific, Waltham, MA, USA). Tissue samples were cut at 5 µm thickness (Rotary Microtome CUT 4055, Microtec, Walldorf, Germany) and mounted on slides (Paraffin section mounting bath, Electrothermal, Staffordshire, UK). Two slides were prepared from each sample leaving 500 µm distance between sampling points. Slides were stained with hematoxylin and eosin stain (Leica Autostainer XL, Leica CV 5040 Coverslipper, Wetzlar, Germany). The images from each slide were captured by QImaging MicroPublisher six color camera (Teledyne QImaging, Surrey, Canada) using OCULAR^TM^, advanced scientific camera control software (Digital Optics Limited, Auckland, New Zealand) fixed to a Leica DMRBE fluorescent microscope (Leica Microsystems, Wetzlar, Germany) under 5 × 10 magnification at Manawatu Microscopy and Image Center, Massey University, Palmerston North, New Zealand. FIJI ImageJ software [17] was then used to measure rumen histomorphometry. Rumen papillae length, width, and thickness of the muscular layer of all complete rumen papillae across each image captured were measured. The number of papillae was noted, the surface length of papillae was measured as described by Dieho et al. [19], and the respective straight length of rumen tissue in the images was measured (Figure 2).

### 2.6. Calculations

Papillae density of the rumen wall was calculated as the number of papillae divided by the respective length of rumen tissue. Papillae surface length per unit of rumen tissue length was calculated as the measured papillae surface length divided by the respective straight rumen tissue length (Figure 2). Thickness of the rumen wall was calculated as the summation of mean papillae height and the thickness of muscular layer of the rumen wall measured from histology slides.

### 2.7. Statistical Analysis

Three lambs out of 27 were excluded from statistical analysis of VFA content, rumen histology, and ultrasound image analysis due to health issues (1 and 2 from HFP57 and LFP42 treatments, respectively). In addition, two lambs from the HFP42 treatment, which were not deprived of feed 12 h before slaughter, were excluded from the VFA and correlation analyses between dietary parameters and rumen development parameters, as their VFA values would have been affected by the different treatments.

Volatile fatty acid content of rumen fluid samples was analysed using a linear model with treatment as a fixed effect (Proc GLM, SAS 9.4, Cary, NC, USA [20]), considering an individual lamb as the experiment unit. Papillae height, width, rumen wall muscle layer thickness, papillae density, and papillae surface length per rumen tissue length were analysed using a linear model with treatment, rumen tissue sampling sites, and their interaction as a fixed effect (Proc mixed, SAS 9.4, Cary, NC, USA [20]) after log (base 10) transformation of the data. Rumen dorsal wall thickness measured by ultrasound scanning was analysed using a linear model with treatment as a fixed effect (Proc GLM, SAS 9.4 [20]), and lamb and replicate ultrasound image were nested within treatment. Differences (*p* < 0.05) were identified, where appropriate, using the least significant difference (LSD) mean comparison test.

An overall summary of pellet, lucerne chaff and nutrient intakes, empty rumen weight, and rumen volume data of all lambs from the study that was used for correlation analysis is presented in Table 2. A detailed analysis of those parameters is presented in Herath et al. [15]. Correlations between ADF, NDF, ME, CP, organic matter, pellet and lucerne chaff intakes, rumen morphology development parameters (empty rumen weight, rumen papillae length, papillae width, and rumen wall muscular layer thickness), and metabolic development parameters (VFA content of rumen fluid) were estimated using Proc CORR, SAS 9.4 Cary, NC, USA [20].

Correlations between total dry matter, ME, CP, organic matter, ADF, NDF, and hemicellulose intakes from pellet and lucerne chaff and the first two estimated principal components for rumen morphology and metabolic development parameters were analysed using Proc CORR, SAS 9.4 (Cary, NC, USA) [20].

Correlations between the thickness of dorsal rumen wall recorded from ultrasound image analysis at seven weeks of age and rumen papillae height, rumen wall thickness, and muscular layer thickness at the dorsal site as recorded from histology image analysis (at slaughter) were estimated using Proc CORR, SAS 9.4 (Cary, NC, USA) [20].

## 3. Results

### 3.1. Volatile Fatty Acid Content of Rumen Fluid

The n-butyric content of rumen fluid at slaughter was greater (*p* < 0.05) in both LFP42 and HFP42 treatment lambs compared to HFP57 lambs, which did not differ (*p* > 0.05, Table 3). The n-valeric content of rumen fluid at slaughter was greater (*p* < 0.05) in LFP42 lambs compared to both HFP57 and HFP42 lambs, which did not differ (*p* > 0.05). The n-caproic content of rumen fluid at slaughter tended to be higher (*p* = 0.06) in LFP42 lambs compared to both HFP57 and HFP42 lambs. The remaining VFA measures did not differ (*p* > 0.05) between treatments.

### 3.2. Rumen Morphology

#### 3.2.1. Histology

The average number of measurements recorded (mean ± SD) for rumen papillae length, width, and muscular layer thickness from dorsal rumen site per lamb was 22 ± 6, 22 ± 7, and 26 ± 7, respectively. From the ventral rumen site the numbers of measurements (mean ± SD) per lamb were 31 ± 10, 31 ± 10, and 27 ± 7, respectively. There was a significant effect of sampling site (Table 4), whereby papillae on the dorsal rumen wall were shorter but wider than those on the ventral rumen wall. Papillae density (number of papillae per cm) was also lower at the dorsal sampling site, whilst dorsal muscle layer thickness was greater than that of the ventral rumen samples. The ratio between the length of papillae outer boundary of the stratum corneum and corresponding straight length of rumen tissue (PSL:STL) was greater (*p* < 0.05) on the ventral site of the rumen.

There was a significant (*p* < 0.05) interaction between rearing regimen/treatment and site of rumen tissue sampling for papillae width, papillae density, and muscle layer thickness (Table 4). Whilst dorsal rumen papillae were significantly (*p* < 0.05) wider than ventral rumen papillae for HFP57 lambs, papillae width was comparable between dorsal and ventral rumen sites for those lambs weaned early; HFP42 and LFP42, and also similar to papillae width at dorsal rumen site of HFP57 lambs. Consequently, papillae density (number of papillae per cm) at the dorsal sampling site was significantly (*p* < 0.05) lower than that at the ventral site for HFP57 lambs. Papillae density was comparable between dorsal and ventral rumen sites for those lambs weaned early, HFP42 and LFP42, and also similar to papillae density at dorsal rumen site of HFP57 lambs. The dorsal rumen of HFP57 lambs had a thicker (*p* < 0.05) muscle layer compared to both the dorsal and ventral rumen sampling sites of HFP42 and LFP42 lambs and ventral sites of HFP57 lambs.

Papillae height, percentage of papillae longer than 500 µm, and the ratio between the length of papillae outer boundary of the stratum corneum and corresponding straight length of rumen tissue (PSL:STL) did not differ between treatments (Table 4). Papillae height, percentage of papillae longer than 500 µm, and the PSL:STL ratio were greater (*p* < 0.05) in the ventral site of the rumen than dorsal site.

#### 3.2.2. Rumen Wall Thickness as Measured by Ultrasound Scanning

**Figure 3:**
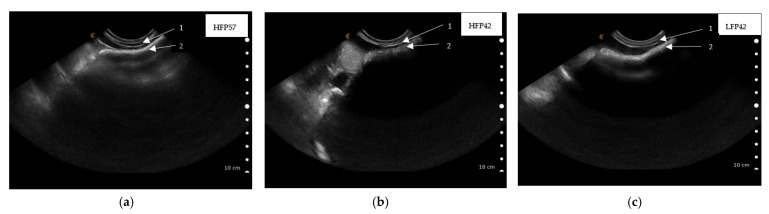
Ultrasound scanned images showing rumen wall thickness captured caudal to the last rib by placing the Clarius ultrasound scanner (3.0–7.0 MHz) parallel to the last ribs of seven-week (49 ± 1.2 d)-old lambs ((**a**) HFP57, high fibre concentrate pellet and milk feeding to 57 d of age; (**b**) HFP42, high fibre concentrate pellet and weaning at 42 d of age; (**c**) LFP42, low fibre concentrate pellet and weaning at 42 d of age) lying on its right side, 1. Abdominal wall, 2. Rumen wall.

Rumen dorsal wall thickness of lambs at five weeks (36 ± 1.2 d) of age, as captured by ultrasound scanning, did not differ between treatments (Table 5). Rumen wall thickness of lambs at seven weeks (49 ± 1.2 d) of age was greater (*p* < 0.05) in both HFP42 and LFP42 lambs compared to HFP57 lambs (Table 5, Figure 3). The variations in lambs within treatment and ultrasound scanned images captured from the same lamb were significant (*p* < 0.05) for thickness of the rumen wall at both five and seven weeks (Table 5).

There were no significant correlations between rumen wall thickness measured via ultrasound at 36 ± 1.2 d and papillae height at dorsal site (r = 0.03, *p* = 0.89), muscle layer thickness at dorsal site (r = 0.34, *p* = 0.10), and rumen wall thickness (r = 0.34, *p* = 0.11) obtained from histology measurements recorded at 57 d of age. There was a significant positive correlation (r = 0.42, *p* = 0.04) between rumen wall thickness measurement obtained from ultrasound scanned images captured at 49 ± 1.2 d of age and the papillae height of dorsal rumen site, measured using histology slides prepared from samples collected at slaughter (56 ± 2.1 d of age). There were no significant correlations between rumen wall thickness measurements obtained from ultrasound scanned images (49 ± 1.2 d of age) and muscle layer thickness (r = −0.23, *p* = 0.27) or thickness of rumen wall at dorsal site (r = −0.04, *p* = 0.87) measured from histology slides based on samples collected at slaughter.

### 3.3. Correlations between Dietary Factors and Rumen Development Parameters

The correlation analysis was carried out between the dietary intake and rumen development using data generated in the study of Herath et al. [15]. Pellet intake of lambs varied between treatments, where HFP42 and LFP42 lambs had greater pellet intake compared to late-weaned lambs (Table 2). Further, there were variations in the pellet intake of individual lambs within the treatment. Consequently, the CP, ME, and organic matter intakes from pellet and lucerne chaff of lambs fluctuated within a huge range (Table 2).

#### 3.3.1. Morphology Development

**Figure 4:**
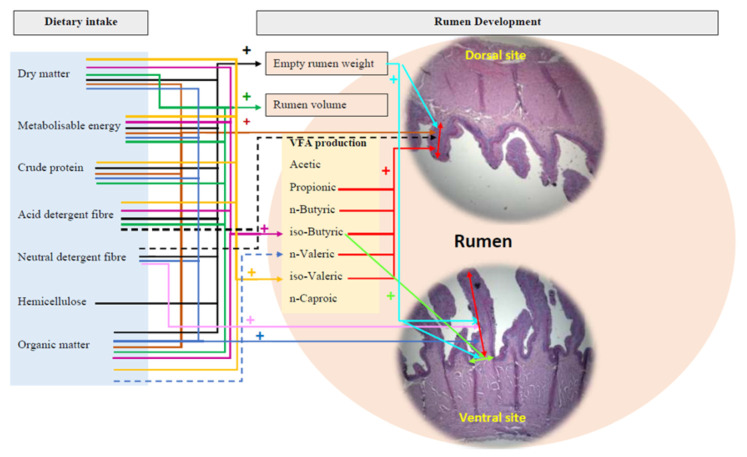
Schematic diagram of correlations between dietary factors and rumen development parameters of artificially reared lambs given three rearing treatments (significant (*p* < 0.05) positive influence and tended positive influence). The parameters combined into a same-colour line influence the respective rumen development parameter (e.g., all parameters joined to the black-coloured solid line positively influenced the empty rumen weight).

Figure 4 shows the significant positive correlations and tendencies in correlations between dietary factors and rumen development parameters. Empty rumen weight had significant (*p* < 0.05) positive correlations with ME, CP, total and pellet dry matter, organic matter intakes from pellets and lucerne chaff, ADF, NDF, and hemicellulose intakes (Table 6, Figure 4). Rumen volume had positive significant (*p* < 0.05) correlations with the ME, CP, total dry matter, organic matter intakes from pellets and lucerne chaff, and ADF intake. Rumen papillae height of both the dorsal and ventral rumen sites had positive significant (*p* < 0.05) correlations with ME, CP, organic matter intake from pellets and lucerne chaff, and dry matter intakes (Table 6, Figure 4).

Empty rumen weight had positive significant correlation with papillae height at the ventral (r = 0.46, *p* = 0.03) and dorsal sites (r = 0.52, *p* = 0.01), and papillae width at the ventral site (r = 0.47, *p* = 0.03) of the rumen (Figure 4).

#### 3.3.2. Metabolic Development

Iso-butyric and iso-valeric contents of rumen fluid had positive significant (*p* < 0.05) correlations with the ME, ADF, dry matter, and organic matter intakes from pellets and lucerne chaff (Table 7, Figure 4). Iso-valeric contents of rumen fluid had positive significant (*p* < 0.05) correlations with the CP intake (Table 7, Figure 4). There were no significant correlations (*p* > 0.05) between all the other dietary and rumen metabolic development parameters (Table A1).

#### 3.3.3. Morphology vs. Metabolic Rumen Development

Papillae height at the dorsal rumen site had significant (*p* < 0.05) positive correlations with propionic (r = 0.49), iso-butyric (r = 0.48), n-butyric (r = 0.61), iso-valeric (r = 0.42), and n-valeric (r = 0.48) contents of rumen fluid (Figure 4). Papillae width at the ventral rumen site had a significant (*p* < 0.05) positive correlation with iso-butyric (r = 0.43) content of the rumen fluid. All the other rumen physical development parameters did not significantly (*p* > 0.05) correlate with the volatile fatty acid content of the rumen fluid (Table A2).

## 4. Discussion

The objective of this study was to investigate the effect of three rearing regimens on rumen development of lambs reared artificially.

### 4.1. Volatile Fatty Acid Content of Rumen Fluid

Lambs weaned early (at 42 d of age) had greater n-butyric content in their rumen fluid at 57 d of age irrespective of their pellet fibre level (HFP vs. LFP). Early weaned lambs consumed more pellets than their unweaned counterparts (HFP57 lambs) [15]. The increased pellet intake is likely responsible for the greater n-butyric content observed. Additionally, the low fibre pellet fed to LFP42 lambs contained 7.5% skim milk powder, which is a source of lactose. Inclusion of lactose in the diet is known to increase butyric acid content of rumen fluid in both adult sheep [21] and cows [22,23,24]. Therefore, the higher pellet intake and inclusion of milk powder in pellets fed to LFP42 lambs would both have contributed to the higher rumen fluid n-butyric acid content compared to HFP57 lambs.

The increased fibre content of the high fibre pellets could have increased acetic acid in the rumen fluid of HFP42 lambs compared to LFP42 lambs. High fibre diets have been reported to favour the production of acetic acid [25]. Acetic and butyric acid are interconvertible to a considerable extent, and acetic and butyric acids show little incorporation into propionic acid [26]. It has been reported that 61% of butyric acid carbon is in equilibrium with approximately 20% of acetic acid in sheep [26]. Hence, the higher n-butyric acid content in HFP42 lambs could be due to the maintenance of the butyric and acetic acid equilibrium in the rumen. Although HFP57 lambs were also fed the high fibre-containing diet, they had lower n-butyric acid due to their lower pellet intake compared to HFP42 lambs. Therefore, the overall higher pellet intake of both HFP42 and LFP42 lambs, the high fibre content of the high fibre pellets, and the inclusion of lactose in the low fibre pellets, likely account for the greater n-butyric acid content in rumen fluid of HFP42 and LFP42 lambs in the present study.

Although comprising a smaller proportion of the VFA pool, branched-chain fatty acids (BCFA: iso-butyrate and iso-valerate) are vital growth factors for cellulolytic bacteria, which degrade the structural carbohydrates in feed and are produced from deamination of valine and leucine by proteolytic bacteria [2,27]. The CP intake of both HFP42 and LFP42 lambs from pellets was higher compared to HFP57 lambs. However, the iso-butyric and iso-valeric acid content of rumen fluid did not differ between treatments, suggesting that net turnover of these BCFA was not impacted by diet or weaning age.

The LFP-fed lambs had higher n-valeric content in their rumen fluid than HFP-fed lambs, irrespective of weaning age. The n-valeric acid in rumen fluid is synthesised from propionyl-CoA and acetyl-CoA, as a product of rumen fermentation [28]. Similarly to n-butyric [21], n-valerate concentration of rumen fluid in cows can be increased by feeding lactose [22,24]. The low fibre pellets fed to LFP42 lambs contained lactose (in the form of skim milk powder), which was absent in high fibre pellet. Thus, the higher n-valeric acid content in LFP42 lambs compared to HFP42 and HFP57 lambs may be explained by the presence of milk powder in their pellets.

In the presence of BCFA, n-valeric acid satisfies the straight carbon chain requirement of cellulolytic rumen bacteria, *Bacteroides succinogenes* [29,30] and n-caproic is required for growth of *Bacteraides succinogenes* [30]. Further, n-valerate, iso-butyrate, and iso-valerate levels are significantly correlated with the abundance of rumen bacterial genera: *Ruminococcaceae_NK4A214*, *Erysipelotrichaceae_UCG.004*, *Olsenella*, *Rikenellaceae_RC9_gut_group*, *Syntrophococcus*, *Prevotellaceae_UCG.001*, *Treponema_2*, *Megasphaera*, *Succinivibrio*, *Prevotella_1* [31]. Higher n-valeric and a tendency for higher n-caproic content in rumen fluid was observed in LFP42 lambs, compared to both HFP42 and HFP57 lambs. The low fibre-fed LFP42 lambs may have had a lower proportion of cellulolytic bacteria in their rumens compared to the high fibre-fed lambs, potentially causing reduced utilisation of n-valeric and n-caproic acids. There is limited research on the impact of dietary fibre level on branched- and straight-chain fatty acid content of young lambs’ rumen fluid and the impact this may have on rumen bacterial composition and rumen function. Future research examining rumen bacteria composition and gene expression would better elucidate the impact of altered fatty acid composition on rumen function.

### 4.2. Rumen Morphology

Rumen morphology parameters, measured histologically, were influenced by an interaction between treatment and rumen tissue sampling site. The HFP57 lambs had reduced papillae width and density at the ventral site compared to the other treatments, suggesting that HFP57 lambs may have a lower surface area at the ventral rumen site for nutrient absorption. The papillae height, percentage of papillae longer than 500 µm, and PSL/STL in both the dorsal and ventral rumen sites were not influenced by treatment, but those parameters were greater for ventral rumen tissue compared to dorsal rumen tissue irrespective of rearing treatment. Hynd [12] reported greater papillae development in the ventral sac of the rumen and proposed that it allowed greater VFA absorption compared to the dorsal sac, as ruminal fluid is in greater contact with the rumen ventral wall compared to the dorsal wall. This is consistent with the increased rumen morphology development (papillae height, percentage of papillae longer than 500 µm, and PSL/STL) observed on the ventral rumen tissue from all lambs in the present study.

Ultrasonographic scanning is a real-time and non-invasive method [32] that has potential to be used to follow rumen development during the transition from non-ruminant to ruminant phase of young ruminants. The lack of differences observed via ultrasound at 36 d suggests that prior to early weaning, rumen development in HFP57, HFP42, and LFP42 lambs was similar. Both HFP42 and LFP42 lambs, early weaned off milk at 42 d of age, had increased rumen dorsal wall thickness at day 49. The early weaned lambs likely had thicker dorsal rumen walls due to their increased solid feed intake positively impacting their rumen development. The positive correlation between rumen wall thickness measured via ultrasound at 49 d of age and papillae height at the dorsal site recorded at slaughter (57 d of age) suggests that the rumen image visualised via ultrasound could be used as an indicator of papillae growth. Additional research is warranted on ultrasonographic examination of morphological changes in the rumen, such as size and wall thickness in different sacs of the rumen, at different ages, adapted feeding programs, early weaning strategies, and rearing systems.

### 4.3. Correlations between Dietary Factors and Rumen Development

Nutrients from milk replacer are directed to the abomasum through the oesophageal groove, thereby bypassing the rumen in bottle-fed lambs [33], and thus milk feeding has little effect on the rumen development [34]. Therefore, dry matter and nutrient intake from pellets and lucerne chaff were only considered for the correlation analyses between dietary factors and rumen development parameters. It is worth noting that due to the treatment effect of early weaning, there was significant variation in the dry matter intake from pellets, CP, ME, and organic matter intake data used for this correlation analysis. The pellet intake of lambs varied between treatments, whereby early weaned lambs had greater pellet intake compared to lambs that continued to be offered milk. Further, there was variation in pellet intake of individual lambs within the treatments.

All VFA in the present study, except acetic and n-caproic acids, positively influenced rumen papillae height at the dorsal sampling site. This is similar to other studies, which found that both the n-butyric acid content of rumen fluid and the n-butyric acid absorption level impacted rumen papillae development of young goats [35]. Moreover, butyric acid reduces the rate of apoptosis of cells [36], leading to increased papillae growth. This likely explains the significant positive correlations observed between papillae height at the dorsal rumen site and both iso- and n-butyric acid content of rumen fluid. Propionic acid has also been found to enhance rumen papillae growth [4,34], which is consistent with the positive correlations observed between propionic acid and rumen papillae development in the present study.

Dry matter, ADF, CP, ME, and organic matter intakes were positively correlated with the iso-valeric acid content of rumen fluid. All these dietary factors, except CP intake, were also positively correlated with iso-butyric content. Iso-valeric and iso-butyric acids are produced as an intermediate product of deamination of proteins by microbes. The dry matter and nutrient intakes from solid feed provide the nutrients required for microbial growth in the rumen. Thus, the observed positive correlations between nutrient intake and iso-valeric and iso-butyric acid content in the rumen fluid are likely due to the influence of nutrient and feed intake on microbial activity in the rumen.

Papillae height at the ventral sampling site of the rumen was positively correlated with dry matter, ME, CP, NDF, and organic matter intakes. Further, papillae height at the dorsal sampling site was positively correlated with dry matter, ME, CP, and organic matter intakes, while ADF and NDF intakes tended to influence dorsal rumen papillae height. These correlations are likely the result of the positive effect dietary intake has on VFA production by microbes and the positive effects the generated VFA have on rumen papillae development. However, it is unclear whether there is any direct influence of nutrient intake on rumen papillae development. Baldwin and Connor [4] reported that typically, studies do not provide adequate details on the direct nutrient-gene interactions related to regulatory pathways of rumen development. Thus, future research is needed to better understand the molecular and nutrient-direct interactions on the rumen development process.

Empty rumen weight was influenced positively by dry matter, ME, CP, ADF, NDF, hemicellulose, and organic matter intakes. Further, papillae height and width at the ventral sampling site and papillae height at the dorsal sampling site had positive correlations with empty rumen weight. The increased VFA production in the rumen with nutrient intake likely improved rumen papillae development and could result in a heavier empty rumen. This suggests that empty rumen weight is both directly and indirectly affected by the nutrient intake from solid feed. However, the mechanism(s) of the direct effect(s) of nutrient intake on the rumen weight require further investigation. Whilst the effect of rumen weight on the digestive and absorptive capacity of the rumen are not well understood [4], the present study showed that rumen mass was positively influenced by papillae height. This increase in the nutrient-absorptive surface area would impact positively on the nutrient-absorption capacity of the rumen. Empty rumen volume was influenced positively by dry matter, ME, CP, ADF, and organic matter intakes. An increase in the bulk intake of feed or high fibre content improves rumen musculature and subsequently increases rumen volume [37]. In the present study, dry matter, ME, CP, ADF, and organic matter intake influenced the rumen volume, but not NDF and hemicellulose intake. In summary, empty rumen weight and volume are affected by the nutrient intake from solid feed, suggesting dry matter intake from solid feed and solid feed composition alter the rumen development of lambs during their early life stage.

## 5. Conclusions

In conclusion, rumen fermentation was improved in lambs early weaned at 42 d of age and by feeding of low fibre pellets. Early weaning improved rumen dorsal wall thickness compared to milk feeding to 57 d of age. Papillae development was not uniform over the rumen luminal surface and was influenced by pellet fibre content and the age of the lambs at weaning. Nutrient intake from solid feed influenced volatile fatty acid production and both nutrient intake and volatile fatty acid production impacted rumen physical development.

These results suggest that both lamb diet composition and age at weaning influence rumen function and physical development. Overall, early weaned lambs fed low fibre pellets appear to have greater nutrient utilisation from solid feed post-weaning. Further research is necessary to confirm if this rearing regimen reduces post-weaning growth due to rumen immaturity and/or improves post-weaning growth due to improved feed efficiency. Additionally, research is required to examine whether early weaning and diet composition of lambs reared artificially influence rumen microbial composition and gene expression pathways related to rumen function and development.

## Figures and Tables

**Table 1:** Pellet composition in fresh matter basis (Adapted from Herath et al. [15]).

Ingredient	Low Fibre Concentrate Pellet (LFP)	High Fibre Concentrate Pellet (HFP)
Barley, g/kg	0.270	0.390
Broll, g/kg ^1^	0.000	0.351
Soya bean meal, g/kg	0.225	0.218
Wheat, g/kg	0.389	0.000
Molasses, g/kg ^2^	0.030	0.030
Skim milk powder, g/kg	0.075	0.000
Limestones, g/kg	0.010	0.010
Sheep premix, g/kg ^3^	0.001	0.001

^1^ Broll is a mixture of wheat bran and wheat pollard, crude protein 153 g/kg, and neutral detergent fibre 359 g/kg; ^2^ Source of molasses is sugar beet; ^3^ Cobalt 0.2 g/kg, Iodine 0.2 g/kg, Magnesium 0.14 g/kg, Selenium 0.04 g/kg, Sodium 0.14 g/kg, Zinc 4 g/kg, and vitamin E 1 IU/g.

**Table 2:** Overall means, standard deviation (SD), and range for pellet, lucerne chaff and nutrients intakes, empty rumen weight, and rumen volume of the lambs included in the study (DMI, dry matter intake; ME, metabolisable energy; CP, crude protein; ADF, acid detergent fibre; NDF, neutral detergent fibre).

Parameter	Mean (*n* = 22)	SD	Range
Total DMI from pellets, kg	7.60	3.31	1.76–12.98
Total DMI from lucerne chaff, kg	0.29	0.16	0.02–0.58
Total DMI, kg	7.89	3.37	2.01–13.47
Total ME intake, MJ	99.17	42.72	24.74–171.10
Total CP intake, g	1616.97	697.34	404.56–2800.56
Combined CP:ME intake, g/MJ	16.31	0.08	16.19–16.38
Organic matter intake from pellets and lucerne chaff ^1^, kg	7.45	3.19	1.90–12.74
Total ADF intake, g	661.24	287.05	197.08–1254.23
Total NDF intake, g	1698.45	825.25	525.26–3380.44
Total hemicellulose intake ^2^, g	1037.21	547.57	312.37–2126.21
Empty rumen weight, g	238.46	68.10	125.30–404.60
Rumen volume, mL	1099.00	327.88	552.59–1917.81

^1^ Calculated as total dry matter intake from pellet and lucerne chaff minus ash intake of respective feedstuff; ^2^ Calculated as total NDF intake minus total ADF intake.

**Table 3:** Effect of three lamb-rearing treatments (HFP57, high fibre concentrate pellets and milk feeding to 57 d of age; HFP42, high fibre concentrate pellets and early weaning at 42 d of age; LFP42, low fibre concentrate pellets and early weaning at 42 d of age) on volatile fatty acid (VFA) content of rumen fluid from artificially reared lambs at slaughter (SE, standard error).

Parameter	Treatment LS Means			Pooled SE	*p*-Value
	HFP57 *	HFP42 *	LFP42 *		
Acetic, mg/g	0.85 (1.41)	0.78 (1.29)	0.93 (1.56)	0.100	0.33
Propionic, mg/g	0.38 (0.51)	0.38 (0.52)	0.42 (0.57)	0.041	0.48
n-Butyric, mg/g	0.13 ^a^ (0.15^)^	0.17 ^b^ (0.20)	0.19 ^b^ (0.22)	0.021	0.019
iso-Butyric, mg/g	0.06 (0.073)	0.07 (0.080)	0.08 (0.090)	0.008	0.18
n-Valeric, mg/g	0.057 ^a^ (0.056)	0.063 ^a^ (0.063)	0.087 ^b^ (0.085)	0.0106	0.018
iso-Valeric, mg/g	0.08 (0.075)	0.08 (0.081)	0.10 (0.097)	0.021	0.29
n-Caproic, mg/g	0.03 (0.026)	0.03 (0.031)	0.05 (0.046)	0.011	0.06
Acetic to propionic ratio ^1^	2.22 (2.75)	1.99 (2.46)	2.19 (2.74)	0.116	0.11
Total VFA, mg/g ^2^	1.58 (22.9)	1.58 (22.6)	1.86 (26.7)	0.172	0.19

^1^ Calculated as acetic acid content divided by propionic acid content of rumen fluid; ^2^ Calculated as summation of all volatile fatty acid contents of rumen fluid; * Individual VFA values in mmol/100 mL and total VFA values in mmol/L are presented in parentheses. ^a, b^ Means in the same row with different superscripts are significantly different (*p* < 0.05).

**Table 4:** Effect of three lamb-rearing treatments (HFP57, high fibre concentrate pellets and milk feeding to 57 d of age; HFP42, high fibre concentrate pellets and early weaning; LFP42, low fibre concentrate pellets and early weaning) on papillae height (µm), width (µm), density (papillae number/cm), muscle layer thickness (µm, MLT), longer (>500 µm) papillae percentage and papillae surface length to straight tissue length (PSL/STL) ratio at the rumen dorsal and ventral sites (D, dorsal rumen site; V, ventral rumen site; SE, standard error).

Variable	Back Transformed Log LSmeans (logLSmeans) ^1^			Pooled Log SE	Back Transformed Log LSmeans (logLSmeans) ^1^		Pooled Log SE	Back Transformed Log LSmeans (logLSmeans) ^1^						Pooled Log SE	*p*-Value		
								Treatment * Rumen Site									
	Treatment				Rumen site			HFP57		HFP42		LFP42					
	HFP57	HFP42	LFP42		D	V		D	V	D	V	D	V		Treat-ment	Rumen Site	Treatment * Rumen site
Papillae height	648.3	748.7	796.5	0.04	649.2 ^a^	817.3 ^b^	0.02	569.4	738.2	675.8	829.5	711.4	891.9	0.04	0.23	<0.0001	0.8651
	(2.8)	(2.9)	(2.9)		(2.8)	(2.9)		(2.8)	(2.9)	(2.8)	(2.9)	(2.9)	(3.0)				
Papillae width	419.6	421.4	437.7	0.02	442.7 ^b^	410.2 ^a^	0.01	478.0 ^b^	368.3 ^a^	414.0 ^ab^	428.7 ^b^	438.3 ^b^	437.1 ^b^	0.02	0.81	0.0006	<0.0001
	(2.6)	(2.6)	(2.6)		(2.7)	(2.6)		(2.68)	(2.57)	(2.62)	(2.57)	(2.64)	(2.64)				
Papillae density	16.2	13.7	14.2	0.04	13.4 ^a^	16.1 ^b^	0.02	13.5 ^a^	19.5 ^b^	13.3 ^a^	14.1 ^a^	13.3 ^a^	15.1 ^a^	0.04	0.33	<0.0001	0.0055
	(1.2)	(1.1)	1.2)		(1.1)	(1.2)		(1.1)	(1.3)	(1.1)	(1.2)	(1.1)	(1.2)				
MLT	1112.8	834.1	874.0	0.04	1006.0 ^b^	864.6 ^a^	0.02	1224.1 ^c^	1011.6 ^a^	913.3 ^a^	761.9 ^b^	910.8 ^ab^	838.7 ^ab^	0.04	0.08	<0.0001	0.0033
	(3.1)	(2.9)	(2.9)		(3.0)	(2.9)		(3.09)	(3.00)	(2.96)	(2.88)	(2.96)	(2.92)				
Papillae > 500 µm% ^2^	63.0	64.5	68.1	0.03	59.0 ^b^	71.9 ^a^	0.02	57.0	69.6	58.8	70.7	61.3	75.7	0.03	0.67	<0.0001	0.9696
	(1.8)	(1.8)	(1.8)		(1.2)	(1.9)		(1.76)	(1.84)	(1.77)	(1.85)	(1.79)	(1.88)				
PSL/STL ^3^	4.0	4.2	4.3	0.04	3.6 ^a^	4.9 ^b^	0.03	3.2	4.9	3.8	4.7	3.7	5.0	0.04	0.82	<0.0001	0.1232
	(0.6)	(0.6)	(0.6)		(0.6)	(0.7)		(0.51)	(0.69)	(0.58)	(0.67)	(0.57)	(0.70)				

^1^ Value in bracket after LS mean represents respective log LS mean. ^2^ Percentage of papillae longer > 500 µm. ^3^ Length of papillae surface/ straight length of rumen tissue. * Interaction effect. ^a, b, c^ Means in the same row within each effect (treatment, rumen site and interaction of treatment, rumen site) with different superscripts are significantly different (*p* < 0.05).

**Table 5:** Effect of three lamb-rearing treatments (HFP57, high fibre concentrate pellets and milk feeding to 57 d of age; HFP42, high fibre concentrate pellets and early weaning at 42 d of age; LFP42, low fibre concentrate pellets and early weaning at 42 d of age) on lamb rumen dorsal wall thickness at five and seven weeks of age as measured from Ultrasound scanned images (SE, standard error).

Lambs Age	Treatment			Pooled SE	*p*-Value		
	HFP57	HFP42	LFP42		Treatment	Lamb ^1^	Image ^2^
5 weeks (36 ± 1.2 d of age), cm	0.25	0.20	0.22	0.04	0.7299	<0.0001	0.0014
7 weeks (49 ± 1.2 d of age), cm	0.33 ^a^	0.48 ^b^	0.56 ^b^	0.05	0.0068	<0.0001	0.0204

^1^ Effect of individual lamb within treatment; ^2^ The effect of replicate Ultrasound scanned image captured from each lamb. ^a, b^ Means in the same row with different superscripts are significantly different (*p* < 0.05).

**Table 6:** Pearson correlation coefficients of dietary factors and rumen physical development parameters of artificially reared lambs exposed to three rearing treatments (DMI, dry matter intake; ME, metabolisable energy; CP, crude protein; ADF, acid detergent fibre; NDF, neutral detergent fibre).

Parameter	Empty Rumen Weight, g	Rumen Volume, mL	Papillae Height-Dorsal, μm	Papillae Height-Ventral, μm	Papillae Width-Dorsal, μm	Papillae Width-Ventral, μm	Muscle Layer Thickness-Dorsal, μm	Muscle Layer Thickness-Ventral, μm
Total DMI from pellets, kg	0.85 **	0.51 *	0.58 *	0.53 *	−0.05	0.30	−0.19	−0.16
Total DMI from lucerne chaff, kg	0.35	0.55 *	−0.03	−0.05	0.31	0.24	0.21	−0.14
Total DMI, kg	0.86 **	0.53 *	0.54 *	0.51 *	−0.03	0.30	−0.18	−0.19
Total ME intake, MJ	0.86 **	0.51 *	0.53 *	0.52 *	−0.03	0.29	−0.18	−0.16
Total CP intake, g	0.86 **	0.51 *	0.53 *	0.52 *	−0.03	0.29	−0.18	−0.15
Combined CP:ME intake, g/MJ	−0.07	−0.36	−0.25	−0.09	0.08	−0.26	0.20	0.23
Organic matter intake from pellets and lucerne chaff ^1^, kg	0.86 **	0.53 *	0.54 *	0.51 *	−0.03	0.30	−0.18	−0.16
Total ADF intake, g	0.81 **	0.46 *	0.39	0.39	0.10	0.20	−0.05	−0.07
Total NDF intake, g	0.77 **	0.34	0.38	0.41 *	0.05	0.13	−0.07	−0.03
Total hemicellulose intake ^2^, g	0.74 **	0.28	0.36	0.42	0.03	0.09	−0.08	−0.004

** Significant at confidence level of <0.0001, * Significant at confidence level of 0.05. ^1^ Calculated as total dry matter intake from pellet and lucerne chaff minus ash intake of respective feedstuff. ^2^ Calculated as total NDF intake minus total ADF intake.

**Table 7:** Pearson correlation coefficients of dietary factors and iso-butyric, and iso-valeric content of rumen fluid of artificially reared lambs exposed to three rearing treatments (DMI, dry matter intake; ME, metabolisable energy; CP, crude protein; ADF, acid detergent fibre; NDF, neutral detergent fibre).

Variable	Iso-Butyric, mg/g	Iso-Valeric, mg/g
Total DMI from pellets, kg	0.54 *	0.49 *
Total DMI from lucerne chaff, kg	0.45 *	0.43 *
Total DMI, kg	0.55 *	0.50 *
Total ME intake, MJ	0.54 *	0.49 *
Total CP intake, g	0.53	0.49 *
Organic matter intake from pellets and lucerne chaff ^1^, kg	0.55 *	0.50 *
Total ADF intake, g	0.48 *	0.45 *
Total NDF intake, g	0.39	0.37
Total hemicellulose intake ^2^, g	0.34	0.32

* Significant at confidence level of 0.05. ^1^ Calculated as total dry matter intake from pellet and lucerne chaff minus ash intake of respective feedstuff. ^2^ Calculated as total NDF intake minus total ADF intake.

## Data Availability

The data presented in this study are included within the article.

## Appendix A

**Table A1:** Pearson correlation coefficients of dietary factors and rumen metabolic development parameters of artificially reared lambs given three rearing treatments (DMI, dry matter intake; ME, metabolisable energy; CP, crude protein; ADF, acid detergent fibre; NDF, neutral detergent fibre).

Parameter	Acetic, mg/g	Propionic, mg/g	n-Butyric, mg/g	n-Valeric, mg/g	n-Caproic, mg/g	Acetic: Propionic
Total DMI from pellets, kg	0.04	0.23	0.33	0.40	0.29	−0.38
Total DMI from lucerne chaff, kg	0.23	0.30	−0.01	0.29	−0.04	−0.04
Total DMI, kg	0.05	0.24	0.32	0.40	0.28	−0.37
Total ME intake, MJ	0.04	0.23	0.32	0.39	0.28	−0.38
Total CP intake, g	0.04	0.23	0.32	0.39	0.27	−0.38
Combined CP:ME intake, g/ MJ	−0.22	−0.19	−0.30	−0.41	−0.32	−0.14
Organic matter intake from pellets and lucerne chaff ^1^, kg	0.05	0.24	0.33	0.41	0.29	−0.37
Total ADF intake, g	0.06	0.23	0.22	0.31	0.15	−0.33
Total NDF intake, g	−0.01	0.16	0.19	0.23	0.12	−0.35
Total hemicellulose intake ^2^, g	−0.04	0.12	0.17	0.18	0.10	−0.35

^1^ Calculated as total dry matter intake from pellet and lucerne chaff minus ash intake of respective feedstuff. ^2^ Calculated as total NDF intake minus total ADF intake.

**Table A2:** Pearson correlation coefficients of rumen metabolic and physical development parameters of artificially reared lambs given three rearing treatments.

Variables	Empty Rumen weight, g	Rumen Volume, ml	Papillae Height-Dorsal, μm	Papillae Height-Ventral, μm	Papillae Width-Dorsal, μm	Papillae Width-Ventral, μm	Muscle Layer Thickness-Dorsal, μm	Muscle Layer Thickness-Ventral, μm
Acetic, mg/g	−0.04	0.20	0.33	0.11	−0.01	0.09	−0.09	0.22
Propionic, mg/g	0.19	0.31	0.49 *	0.23	−0.13	0.28	−0.16	0.19
Iso-Butyric, mg/g	0.35	0.30	0.48 *	0.14	−0.04	0.43 *	−0.14	−0.28
n-Butyric, mg/g	0.22	0.17	0.61 *	0.23	−0.26	0.17	−0.26	0.10
Iso-Valeric, mg/g	0.31	0.24	0.42 *	0.10	−0.05	0.36	−0.11	−0.33
n-Valeric, mg/g	0.28	0.35	0.48 *	0.21	−0.17	0.25	−0.21	0.08
n-Caproic, mg/g	0.07	0.001	0.22	0.33	−0.18	−0.14	−0.05	0.09
Acetic: Propionic	−0.48	−0.12	−0.16	−0.22	0.18	−0.39	0.09	0.20

* Significant at confidence level of 0.05.
